# Congenital Hypogonadotropic Hypogonadism With Novel Pathogenic Variants in FGFR1 and GNRHR

**DOI:** 10.1210/jcemcr/luae254

**Published:** 2025-01-15

**Authors:** Shinta Yamamoto, Hanako Nakajima, Hiroshi Okada, Naoko Nakanishi, Masahide Hamaguchi, Michiaki Fukui

**Affiliations:** Department of Endocrinology and Metabolism, Graduate School of Medical Science, Kyoto Prefectural University of Medicine, Kyoto 602-8566, Japan; Department of Endocrinology and Metabolism, Graduate School of Medical Science, Kyoto Prefectural University of Medicine, Kyoto 602-8566, Japan; Department of Endocrinology and Metabolism, Graduate School of Medical Science, Kyoto Prefectural University of Medicine, Kyoto 602-8566, Japan; Department of Endocrinology and Metabolism, Graduate School of Medical Science, Kyoto Prefectural University of Medicine, Kyoto 602-8566, Japan; Department of Endocrinology and Metabolism, Graduate School of Medical Science, Kyoto Prefectural University of Medicine, Kyoto 602-8566, Japan; Department of Endocrinology and Metabolism, Graduate School of Medical Science, Kyoto Prefectural University of Medicine, Kyoto 602-8566, Japan

**Keywords:** congenital hypogonadotropic hypogonadism, *FGFR1*, *GNRHR*, gene pathogenic variant

## Abstract

Congenital hypogonadotropic hypogonadism (CHH) can cause delayed secondary sexual characteristics and contribute to juvenile osteoporosis, with multiple causative genes having been reported. We treated a 27-year-old man diagnosed with central hypogonadism, presenting with delayed secondary sexual characteristics and juvenile osteoporosis, using bone resorption inhibitors and testosterone therapy. Genetic testing revealed missense variants both in the fibroblast growth factor receptor 1 (*FGFR1*) and gonadotropin-releasing hormone receptor (*GNRHR*) genes, a combination that has not been previously reported. This case represents a CHH caused by a novel combination of gene variants not registered in the human genome mutation database.

## Introduction

Congenital hypogonadotropic hypogonadism (CHH) is a rare disorder, affecting 1.2 to 10 individuals per 100 000 people. It is typically identified by delayed secondary sexual development or cryptorchidism in male children, and in some adults, by reduced libido or osteoporosis [1]. CHH is a genetic disorder, with the first causative gene, *ANOS1*, identified in 1991, and currently, more than 60 related genes have been reported [2]. More than half of CHH patients present with olfactory dysfunction, leading to a diagnosis of Kallmann syndrome (KS), while those without olfactory impairment are termed *normosmic CHH*.

## Case Presentation

The patient is a 27-year-old man who had been followed in pediatric care for autism since childhood. He underwent surgery for a ventricular septal defect at age 1 and cryptorchidism at age 2. He exhibited no other congenital anomalies, such as cleft lip or limb malformations. At age 17, he suffered fractures of the right femoral neck and L1 vertebra due to a fall and was treated conservatively with compression hip screw surgery and lumbar compression band therapy. In the year X-1, he reported back pain, and lumbar x-rays revealed multiple vertebral fractures. Dual-energy x-ray absorptiometry showed young adult mean (YAM) value of 39% for the lumbar spine and 57% for the femur, leading him to seek further evaluation for severe juvenile osteoporosis.

## Diagnostic Assessment

The patient's height at the first visit was 166 cm, and his weight was 61 kg. The parents’ height was 170 cm, but the predicted height of 178.5 cm was not achieved [3]. Delays in mental development such as autism, as well as delays in physical development such as head control and sitting, were observed. Physical examination revealed the absence of pubic hair and voice change, with Tanner stage 1 secondary sexual characteristics. Additionally, no abnormalities were observed in limb deformities, heart sounds, or respiratory sounds. Blood tests were notable for low levels of luteinizing hormone (0.14 mIU/mL; reference interval, 2.2-8.4 mIU/mL), follicle-stimulating hormone (0.61 mIU/mL; reference interval, 1.8-12.0 mIU/mL), and free testosterone (0.5 pg/mL; reference interval, 7.6-23.8 pg/mL). Other pituitary functions, including adrenal and thyroid functions, were normal. The patient did not report any noticeable olfactory impairment, and head magnetic resonance imaging to evaluate olfactory bulb formation could not be conducted due to the patient's inability to remain still. Similarly, resting blood tests and endocrine challenge tests were not feasible. Based on these findings, the patient was diagnosed with normosmic hypogonadotropic hypogonadism and secondary osteoporosis.

Genetic testing (performed by Kazusa DNA Research Institute) revealed a nonsense variant in codon 772 of the fibroblast growth factor receptor 1 (*FGFR1*) gene, changing glutamine at position 258 to a stop codon. Additionally, a missense variant in codon 772 of the gonadotropin-releasing hormone receptor (*GNRHR*) gene was found, changing glycine at position 172 to arginine. These findings led to a diagnosis of hypogonadotropic hypogonadism and secondary osteoporosis caused by *FGFR1* and *GNRHR* gene pathogenic variants. Both the *FGFR1* and *GNRHR* genes showed heterozygous genetic abnormalities.

## Treatment

Treatment was initiated with intramuscular testosterone enanthate 250 mg/week for hypogonadism, along with oral alfacalcidol 0.5 μg/day and minodronate 50 mg/day for osteoporosis. Due to difficulties with frequent visits, treatment with testosterone enanthate was initiated at 250 mg/week instead of 125 mg/week. No side effects, such as liver dysfunction, were observed. However, acne developed after starting the treatment, and the dosage was reduced to 125 mg/week after 5 months.

## Outcome and Follow-up

After 30 days of treatment, signs of secondary sexual development, such as facial and back acne, increased body hair, and voice change, were observed, and the lumbar spine YAM value improved from 39% to 55%.

## Discussion

We encountered a case of a 27-year-old man diagnosed with central hypogonadism due to delayed secondary sexual development and juvenile osteoporosis. Congenital central hypogonadism is believed to be caused by genetic pathogenic variants, with genes such as *ANOS1*, *FGFR1*, *FGF8*, *PROKR2*, and *PROK2* being implicated [4]. While initially thought to be a monogenic disorder [5, 6], recent advancements in genomic medicine have identified multiple causative genes in patients with congenital central hypogonadism, and the concept of “oligogenicity,” in which pathogenic variants in 2 or more genes are identified in the same patient, has been reported [7].

**Figure 1.**
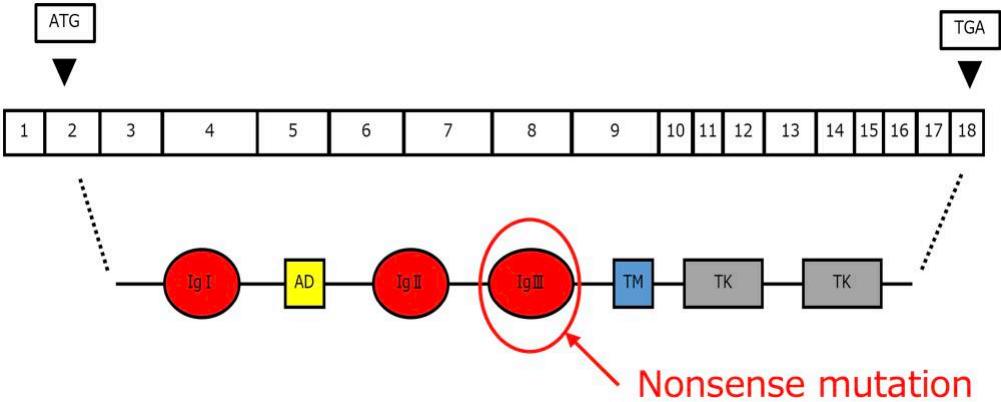
Structure and variants of the *FGFR1* gene. A nonsense mutation was identified in part of the gene for the ligand's IgIII domain. FGFR1, fibroblast growth factor receptor 1.

The *FGFR1* gene, located on 8p11.22, plays a role in the extension of gonadotropin-releasing hormone neurons from the olfactory placode to the brain and the formation of the olfactory bulb. It was the second gene identified as associated with KS and follows an autosomal dominant inheritance pattern. Numerous variants have been reported in this gene, which consists of 18 exons coding for the FGFR1 protein [8]. This protein includes extracellular and intracellular domains and forms dimers to exhibit signaling activity. The glutamine at position 258, where the pathogenic variant was found, is part of the ligand's IgIII domain responsible for dimer formation, and the nonsense variant likely resulted in its inactivation (Fig. 1).

**Figure 2.**
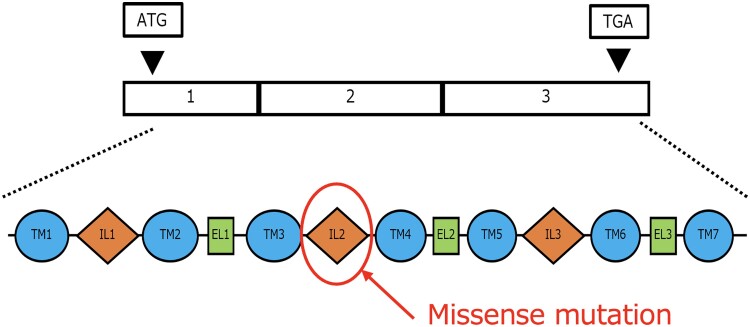
Structure and variants of the *GNRHR* gene. A missense mutation was identified in a part of the gene within the IL2 domain. GNRHR, gonadotropin releasing hormone receptor.

**Figure 3.**
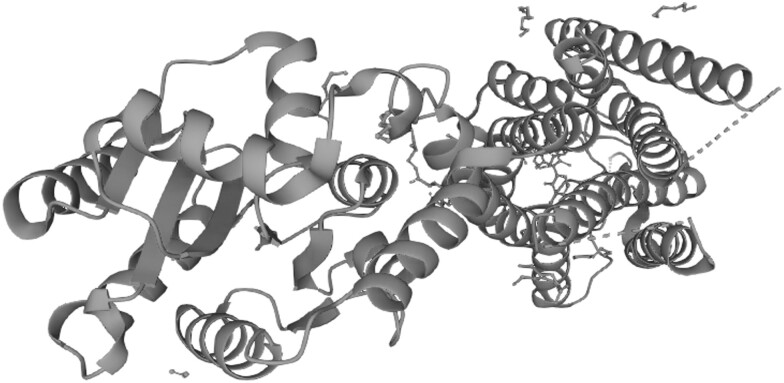
Structural variants of the *GNRHR* gene analyzed using Polymorphism Phenotyping v2.

The *GNRHR* gene, located on 4q21.2, follows an autosomal recessive inheritance pattern and is found on the surface of gonadotropin-releasing hormone–secreting cells in the pituitary gland. Pathogenic variants in this gene account for approximately 10% of cases of normosmic CHH without associated anomalies [9]. The gene consists of 3 exons and codes for a 7-transmembrane domain G protein–coupled receptor, which releases luteinizing hormone and follicle-stimulating hormone on activation (Fig. 2). The clinical significance of the missense variants found in this case was uncertain, so in silico analysis using Polymorphism Phenotyping v2 (http://genetics.bwh.harvard.edu/pph2/) was conducted. The pathogenic variant was deemed “probably damaging,” suggesting that its inactivation contributes to hypogonadism (Fig. 3).

In large-scale variants analyses of KS/normosmic CHH patients, the presence of oligogenic inheritance, in which a pathogenic variant in the *GNRHR* gene is accompanied by variants in other normosmic CHH/KS-related genes, has been reported [10]. In this case, pathogenic variants in 2 genes were identified, both contributing to hypogonadism, leading to a diagnosis of oligogenic CHH. The *FGFR1* gene alone has approximately 900 variants registered in the human genome mutation database, while the *GNRHR* gene has around 200. However, the 2 gene pathogenic variants found in this case are novel and unregistered, suggesting that this is a new genetic variant. As complex genetic patterns involving variants in multiple genes can lead to CHH, as in this case, it is crucial to conduct comprehensive genetic variants analyses using next-generation sequencing targeting known genes.

## Learning Points

- This CHH case reveals oligogenicity with novel coexisting variants in the *FGFR1* and *GNRHR* genes, highlighting the complexity.
- Comprehensive genetic testing using next-generation sequencing is essential for understanding rare genetic disorders like CHH.
- Personalized multidisciplinary treatment based on genetic and clinical features contributes to secondary sexual development and improved bone density in CHH patients.

## Contributors

All authors made individual contributions to authorship. S.Y., N.N., and H.N. were involved in the diagnosis and management of the patient and manuscript submission. H.O., M.H., and M.F. contributed to the interpretation of genetic mutations and the creation of figures. All authors reviewed and approved the final draft.

## Data Availability

Data sharing is not applicable to this article as no data sets were generated or analyzed during this study.

## Abbreviations

CHH: congenital hypogonadotropic hypogonadism
*FGFR1*: fibroblast growth factor receptor 1 gene
*GNRHR*: gonadotropin-releasing hormone receptor gene
KS: Kallmann syndrome
YAM: young adult mean
